# Human Response to Longitudinal Perturbations of Standing Passengers on Public Transport During Regular Operation

**DOI:** 10.3389/fbioe.2021.680883

**Published:** 2021-07-23

**Authors:** Simon Krašna, Arne Keller, Astrid Linder, Ary P. Silvano, Jia-Cheng Xu, Robert Thomson, Corina Klug

**Affiliations:** ^1^ Faculty of Mechanical Engineering, University of Ljubljana, Ljubljana, Slovenia; ^2^ AGU Zürich, Zürich, Switzerland; ^3^ Swedish National Road and Transport Research Institute, Linköping, Sweden; ^4^ Mechanics and Maritime Science, Chalmers University, Gothenburg, Sweden; ^5^ Vehicle Safety Institute, Graz University of Technology, Graz, Austria

**Keywords:** balance recovery, non-collision incidents, public transport, standing passengers, volunteer tests

## Abstract

This study investigates the response of standing passengers on public transport who experience balance perturbations during non-collision incidents. The objective of the study was to analyse the effects of the perturbation characteristics on the initial responses of the passengers and their ability to maintain their balance. Sled tests were conducted on healthy volunteers aged 33.8 ± 9.2 years (13 males, 11 females) standing on a moving platform, facilitating measurements of the initial muscle activity and stepping response of the volunteers. The volunteers were exposed to five different perturbation profiles representing typical braking and accelerating manoeuvres of a public transport bus in the forward and backward direction. The sequence of muscle activations in lower-extremity muscles was consistent for the perturbation pulses applied. For the three acceleration pulses combining two magnitudes for acceleration (1.5 and 3.0 m/s^2^) and jerk (5.6 and 11.3 m/s^3^), the shortest muscle onset and stepping times for the passengers to recover their balance were observed with the higher jerk value, while the profile with the higher acceleration magnitude and longer duration induced more recovery steps and a higher rate of safety-harness deployment. The tendency for a shorter response time was observed for the female volunteers. For the two braking pulses (1.0 and 2.5 m/s^2^), only the lower magnitude pulse allowed balance recovery without compensatory stepping. The results obtained provide a reference dataset for human body modelling, the development of virtual test protocols, and operational limits for improving the safety of public transportation vehicles and users.

## Introduction

The safety of passengers on public transport is a prerequisite for a sustainable transport system, as even minor incidents and frequent discomfort can discourage vulnerable people from using public transport. On public transport vehicles, such as buses and trams, standing passengers are exposed to the risk of injury due to falling during regular trips (so-called non-collision incidents). The risk of falling in a moving vehicle was estimated to be between 0.3 and 0.5 falls per million passenger kilometres (Elvik, 2019). A recent study (Silvano and Ohlin, 2019) found that the circumstances for which passenger falls occur, and the groups typically affected, are different during acceleration and braking. During acceleration and turning from the bus stop, passengers fall after boarding, while attempting to become seated. This affects those aged 65+ and female users in particular, who are also overrepresented among public transport users in these type of non-collision incidents on buses (Kirk et al., 2003; Albertsson and Falkmer, 2005; Björnstig et al., 2005; Halpern et al., 2005; Kendrick et al., 2015; Barnes et al., 2016). In contrast, during braking, falling events typically occur while travelling and affect males, females and different age groups similarly (Silvano and Ohlin, 2019). Apart from age, different body proportions, compositions, and muscle strengths in males and females are important factors when studying and improving traffic safety (Vasavada et al., 2001; Carlsson et al., 2011; Jin et al., 2019). However, some researchers observed no gender-related differences in the response to standing-posture perturbations (De Graaf and Van Weperen, 1997).

Balance is maintained if the centre of mass of the human body is within the base of support—an area projected onto the floor under and between the feet (Maki and McIlroy, 1997). In order to achieve this, three major strategies have been identified: ankle, hip and stepping strategies (Winter, 1995). The ankle and hip strategies, also referred to as fixed-support strategies, are applied during less severe perturbations. Step responses are referred to as a change in support strategies if the centre of mass moves beyond the base of support. All these strategies represent two ends of a continuum of responses that involve a combination of both strategies (fixed-support and change-in-support) with different muscle-activation patterns. In the ankle strategy, the anterior muscles of the lower extremities are typically activated in a distal-to-proximal sequence in response to small forward perturbations, while posterior muscles counteract inertia of the body when a backward perturbation is applied. In more severe perturbations, the hip strategy is evoked, where hip flexors (abdominal muscles, quadriceps) are activated in backward perturbations and hip extensors (lower back, biceps femoris) in forwards perturbations to generate hip torques (Horak and Nashner, 1986; Runge et al., 1999; Blenkinshop et al., 2017). The hip strategy is characterized by longer muscle onset latencies (Torres-Oviedo and Ting, 2007). Generally, muscle onset latencies obtained from electromyography (EMG) were found to be closely correlated with the timing of joint motions (Hwang et al., 2009).

Change-in-support strategies, where a recovery step changes the base of support for stability, are used when fixed-support strategies are no longer effective, which can be the case for the perturbation levels encountered on public transport vehicles. Compensatory stepping is initiated and executed faster than volitional movements (Maki and McIlroy, 1997). The reaction times of the muscles are approximately 90–130 ms, and about one second is needed to retain balance in the case of larger movements (Horak and Nashner, 1986; Winter, 1995; Runge et al., 1999; Simoneau and Corbeil, 2005; Powell and Palacín, 2015). Owings et al. (2001) studied stepping strategies in volunteers standing on a treadmill accelerating to 0.89 m/s in 150 ms. The average reaction time between the onset of the treadmill motion and the recovery step toe-off was estimated to be 0.24 ± 0.03 s for a successful recovery and 0.28 ± 0.05 s for a failed recovery group of older volunteers. The subjects exposed to a high jerk do not have sufficient time to react, even to low acceleration levels. The ability to perform fast and effective compensatory stepping is important for successful balance recovery in response to a standing-posture perturbation—a shorter step initiation and completion time can be related to improved balance (Rogers et al., 2003). Young, healthy adults were reported to mostly use a single recovery step, while for the same balance perturbations, elderly people tend to use multiple stepping, which was also identified as a robust predictor of fall risk in the elderly, particularly in lateral perturbations (Mille et al., 2013). Increasing perturbation intensity requires modifying the fixed-support strategies to a single-stepping or multiple-stepping response (de Kam et al., 2017). Multiple steps can also result in larger displacements of the whole body, particularly the head, implying an increased injury risk from impacting elements of the bus interior (Robert et al., 2007a; Siman-Tov et al., 2019; Zhou et al., 2020).

The shape, magnitude and duration of a perturbation profile can have a significant effect on the standing passenger’s response during non-collision incidents (Robert et al., 2007a; Robert et al., 2007b). A typical bus deceleration (braking) profile is characterized by a rather long magnitude rise time. A vehicle acceleration profile exhibits a sharp initial slope (high jerk), with a gradual decrease of the acceleration magnitude afterwards. For normal bus braking, the reported values of deceleration magnitude ranged from 1.2 to 3.0 m/s^2^ (Kühn, 2013; Kirchner et al., 2014; Schubert et al., 2017). The acceleration magnitudes for bus departures were reported to be 0.8–2.5 m/s^2^, and the jerk magnitude values reported were up to 15.7 m/s^3^ (Brooks et al., 1980; Kühn, 2013; Kirchner et al., 2014; Schubert et al., 2017). The duration of normal acceleration and braking can range from 8.4 to 13.6 s, depending on the velocity change of the vehicle (Kirchner et al., 2014; Schubert et al., 2017). An increased risk of falling is related to the magnitudes of acceleration and jerk that require recovery stepping in response to perturbation, thus exceeding the level of comfort (Powell and Palacín, 2015). Karekla and Fang (2021) proposed a threshold of 1.0–1.5 m/s^2^ for comfortable gait and balance without handrails on a bus during operation.

In addition to field studies (Hoberock, 1976; Brooks et al., 1980; Schubert et al., 2017; Karekla and Tyler, 2018; Karekla and Fang, 2021), laboratory research enabling more controlled conditions has addressed the balance recovery of standing people by exposing volunteers to external perturbations in different settings. The perturbation can be generated in different ways, such as waist-pulls, sudden release of a person held in a tilted position by a rope, and moving platforms (Owings et al., 2001; Hsiao-Wecksler and Robinovitch, 2007; Cyr and Smeesters, 2009; Mille et al., 2013; Bair et al., 2016; Čamernik et al., 2016; Borelli et al., 2019). For practical reasons, the moving platforms and treadmills typically exhibited smaller displacements and durations of platform motion than expected on public transport vehicles (De Graaf and Van Weperen, 1997; Szturm and Fallang, 1998; Carpenter et al., 2005; Tokuno et al., 2010; Kirchner et al., 2014; Sarraf et al., 2014; Zemková et al., 2016; Koushyar et al., 2019).

A series of volunteer tests with standing people on a moving platform was performed by Robert et al. (2007a and Robert et al., 2007b), employing 2.0–10.0 m/s^2^ perturbations of 400-ms duration and different set-up configurations (free-standing, grasping). Comparing the horizontal excursion and the velocity of the head revealed that the volunteers applied different strategies for balance recovery. In a simulation study with a multibody human body model, the time to fall was estimated to be about 2.5 s (Palacio et al., 2009). This suggests that a volunteer test needs to employ perturbations longer than 2.0–2.5 s to fully investigate the potential outcome of a passenger losing balance, but the measurement system must have the resolution to detect nuances in the kinematic responses during 300–400-ms intervals.

Providing experimental data that characterise the response of standing passengers in realistic conditions is necessary to assess the injury risk of standing passengers in different traffic situations. Furthermore, such a dataset is needed for the development of a validated human body model (HBM) for a standing passenger, which can utilize the advantages of numerical simulations for the safety improvements of vehicle designs and operation, in addition to the traditionally recommended measures of prevention (Siman-Tov et al., 2019; Zhou et al., 2020).

The current knowledge of how vehicle motion influences the risk of non-collision incidents is still insufficient to provide guidance to the drivers of today’s buses and trams and to the developers of future autonomous vehicles. In particular, a better understanding of the factors that cause a person to lose balance when faced with a given perturbation is needed. The overall objective of this study was to collect experimental data for the development of a standing passenger HBM as a tool for assessing a passenger’s response to different balance perturbations. A novel test setup for standing-passenger volunteers is introduced and the first analysis of the recorded data from a test series with healthy volunteers is presented. The first objective was to identify how the characteristics of the perturbation pulse affect the initial passenger responses, in particular how passengers react to the direction, magnitude and duration of a balance perturbation resulting from a bus braking or accelerating. The second objective was to understand the consequences of the pulses on different passengers, specifically the possible differences between the initial response of male and female volunteers. The literature identified different demographics and injury scenarios that warrant further investigation.

## Methods

### Test Set-Up

**FIGURE 1:**
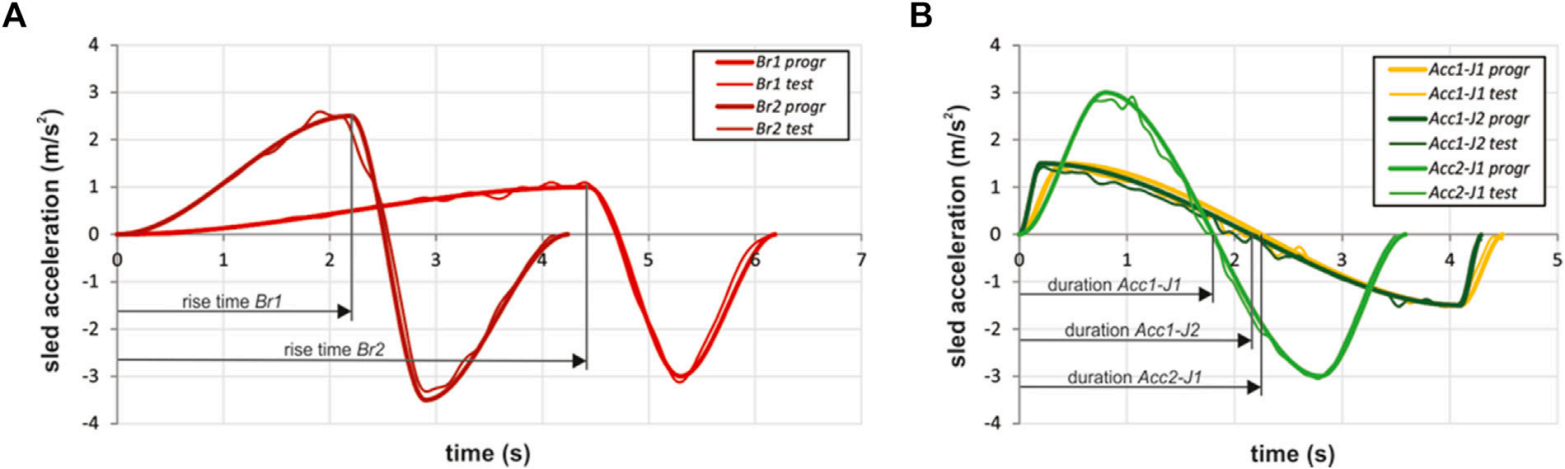
Perturbation pulses applied to the moving platform with standing volunteers, representing braking **(A)** and acceleration **(B)** of a public transport bus; programmed pulses (thick lines), exemplary measured pulses (thin lines). Arrow lines indicate the parts of the pulses used in analysis of the volunteers’ responses.

In this study, 24 instrumented volunteers were exposed to five different perturbation pulses in the forward and backward directions. The tests were conducted on the linear translational platform shown in Figure 1. Two servomotors were used to propel the platform according to predefined motion profiles. The volunteers were perturbed from a stationary position by the motion profile of interest. After the initial perturbation, the platform was brought back to rest. The displacement of the platform during the perturbation and the subsequent deceleration to rest were limited by the range of motion of the test device (5.5 m).

The acceleration profiles were reviewed to define the test pulses that could be used for the volunteer testing in a laboratory. In addition to the literature reviewed, proprietary measurements of urban-bus accelerations were performed during regular service and closed track tests (unpublished in-house experimental data) to estimate the main pulse characteristics. Emergency manoeuvres that substantially exceeded the passengers’ balance thresholds were beyond the scope of the study.

The pulses for the volunteer tests were selected to represent severity levels typically arising during regular travel for non-collision incidents, but greater than the published comfort thresholds. The pulse durations were selected to study the initial response of the participating volunteers, as well as a time frame that captures their balance strategies. Furthermore, the pulse should be long enough to estimate whether the resulting motion of the participant would put a real bus passenger at risk of colliding with the vehicle interior. After the initial pilot tests with volunteers were carried out, the magnitude and duration of the final set of pulses were defined, aiming to have a mix of pulses where volunteers are able to maintain, but also lose their balance.

Each volunteer could experience up to five different perturbation profiles, described in Table 1, representing the typical braking and accelerating manoeuvres of a public transport bus. For the braking pulses *Br1* and *Br2,* two platform acceleration magnitudes were selected (1.0, 2.5 m/s^2^). For the acceleration pulses *Acc1-J1*, *Acc1-J2* and *Acc2-J1*, two magnitudes of acceleration (1.5, 3.0 m/s^2^) were combined with two magnitudes of jerk (5.6, 11.3 m/s^3^) to define five different sled motion profiles. The programmed time profile of the perturbation pulses is depicted in Figure 1 and compared to the sled accelerations measured in a set of pilot trials.

**TABLE 1:** Perturbation profile characteristics.

*Profile name*	Sequence	Magnitude	Rise time	Duration	Jerk	Displacement	Max. Speed
		**m/s^2^**	**s**	**s**	**m/s^3^**	**m**	**m/s**
*Br1*	1	1.0	4.4	4.7	0.3	2.94	2.4
*Br2*	5	2.5	2.2	2.5	1.7	1.82	3.2
*Acc1-J1*	2	1.5	0.4	2.3	5.6	2.65	2.0
*Acc1-J2*	3	1.5	0.2	2.2	11.3	2.58	2.0
*Acc2-J1*	4	3.0	0.8	1.8	5.6	2.69	3.1

The study of the volunteers’ response was limited to the pulse segments denoted as the initial rise time (time to peak) of the braking pulses (Figure 1A) and the duration of the acceleration pulses (Figure 1B), before the sled starts to decelerate in order to bring the platform to a stop. Although longitudinal manoeuvres of the bus could take longer on regular trips, e.g., when braking from or accelerating to cruising travel speed, the pulse segments considered still enabled an analysis of the initial volunteer response to characteristic perturbation pulses. As the bus braking and acceleration pulses were simulated in the same sled direction, a forward-facing volunteer experienced the accelerations similar to a transit passenger facing the direction of travel, whereas the braking pulses were experienced as if the passenger were facing backwards in the vehicle, opposite to the direction of travel. The opposite was true for the backward-facing passenger.

### Volunteers

A total of 24 volunteers participated in the study (13 males and 11 females), representing on average a body weight and height close to a 50th percentile anthropometry (Table 2). The height of the centre of gravity from the ground was estimated using the centre of volume from the 3D scans of the volunteers performed prior to the tests with an infrared scanning device. The volunteers that were recruited (general health was required) were asked if they had any health issues that could affect the balance. Additionally, a participating physician made a quick assessment of each volunteer to confirm the absence of health issues. Considering the age group (younger adults, average), no further tests were performed to assess the volunteers’ capabilities or to profile them. Prior to the tests, the volunteers were familiarized with the scope of tests and signed an informed consent. The design of the study and the consent form were approved by Slovenian National Medical Ethics Committee (application number 0120-63/2019/4).

**TABLE 2:** Basic anthropometric and demographic data for the volunteers (mean ± SD).

	Age	Mass	Height	Centre of mass height
	**years**	**kg**	**cm**	**cm**
11 females	31.6 ± 7.2	64.7 ± 9.9	165.5 ± 6.4	91.2 ± 3.9
13 males	35.5 ± 10.6	86.2 ± 11.8	179.2 ± 5.4	99.2 ± 3.6

The study was focused on free-standing occupants subjected to perturbations in anterior-posterior directions. The volunteers stood on the moving platform with their feet hip-width apart to provide uniform initial conditions for the volunteers. This posture could also represent the standard posture for a standing HBM. Each volunteer experienced two series of perturbations in the following order: 1. *Br1*, 2. *Acc1-J1*, 3. *Acc1-J2*, 4. *Acc2-J1*, 5. *Br2*. During the first series, the test subjects were facing the direction of travel, while for the second series, they were facing backwards. During a series of pre-tests, *Br2* was identified as the most challenging perturbation, with a high magnitude needed to stop the platform due to design limitations (Figure 1A). Therefore, if the participants visibly had trouble withstanding the first four perturbations, *Br2* was omitted for safety reasons. The time between two sequential tests was approximately 3 min. In order to prevent a possible adaptation to the perturbations, the volunteers were not informed about the pulse characteristics and the sequence of application prior to the tests. About 30 s before a test was initiated, the volunteers were instructed to maintain a relaxed free-standing posture on the moving platform as they would as passengers on a bus. To reduce the effect of possible anticipation, no indication was given to when the test was to start. The main switch for controlling the sled was out of sight and no noise from the motors and linear drive was generated when the sled was at rest. If technical difficulties occurred during one or several of the tests, they were repeated at the end of the test series and only the data from the repeated tests were included in the further analyses.

The volunteers wore uniform tight outfits and flexible thin rubber-soled shoes. For safety reasons, a cushion was placed in the location on the platform where a fall could have happened. Additionally, to prevent the volunteers from falling off the platform or hitting the sled frame, they wore a full-body harness and were attached to the moving platform with two ropes. The length of the ropes was adjusted to each individual volunteer so as not to obstruct their motion during an attempt to recover their balance, allowing approximately 1.3 m of horizontal excursion before the harness was deployed.

### Instrumentation

Two high-speed cameras (VEO 640L, Vision Research, Wayne, NJ, United States) captured the volunteer’s motion in the sagittal and frontal planes. The muscle activity was measured using an 8-channel TeleMyo 2400T G2 system (Noraxon, Scottsdale, AZ, United States) for electromyography (EMG) at a 3-kHz sampling frequency. Bipolar Ag/AgCl surface electrodes (Skintact F-301, Innsbruck, Austria) were attached to the lower extremity muscles after the skin surface was shaved and cleaned with a propanol-based solution. The EMG electrodes were placed and fixed bilaterally according to SENIAM recommendations on the rectus femoris (RF), tibialis anterior (TA), biceps femoris (BF) and gastrocnemius medialis (GM).

Body-segment motions were captured with a system of eight cameras Oqus 3+ (Qualisys, Gothenburg, Sweden) tracking 56 passive reflective markers attached to the volunteer’s body at a sampling rate of 200 fps. For measuring the ground-reaction forces, a force plate (HE600600-2k, AMTI, Watertown, MA, United States) was rigidly attached to the moving platform and connected to a LabVIEW data-acquisition card sampling at a 1-kHz frequency using an analogue low-pass filter with a 100-Hz cut-off frequency. The main switch was connected to the trigger providing the synchronisation signal. Additionally, six wearable inertial measurement units (MetaMotionR, MbientLab, San Francisco, CA, United States) were attached to the volunteer’s body segments (lower legs, lower arms, head and pelvis) to track their motion by streaming the accelerometer and gyroscope data at 100 Hz.

### Data Analysis

To study the effect of the pulse characteristics on the initial response of the passenger, tables were generated with variables describing the pulse characteristics like direction and magnitude, as well as the volunteer-response parameters like foot-contact times and EMG reference times. These tables allow for statistical analyses that identify the main and combined effects of the perturbation variables on the volunteers’ responses. The acceleration and braking pulses were analysed independently.

The recorded EMG signals were band-pass filtered with a 4th-order zero-lag Butterworth filter (20–500 Hz), full-wave rectified, and low-pass filtered with a 6th-order zero-lag Butterworth filter with a 6-Hz cut-off frequency. For detecting muscle onset, the band-passed signals were filtered with a low-pass 4th-order zero-lag Butterworth filter with a 50-Hz cut-off frequency. The onset was defined as the first sample of a 50-ms moving-average window exceeding the threshold of 2.5 standard deviations of the EMG signal over the resting period before the initiation of the perturbation (Hodges and Bui, 1996) and was checked visually for each signal measured. The EMG signal processing was performed in Matlab (Natick, United States).

The sequence of events during the balance recovery was identified from the high-speed video recordings, where up to four sequential steps were tracked. The timing of the first frame when the contact between the foot and the ground (the moving platform) was lost was identified as the *contact-off* time, while the time of re-establishing the contact was identified as the *contact-on* time. The difference between *contact-off* and *contact-on* for the same (swing) foot represented the *swing time*. If the volunteer’s motion was restricted by the harness before the end of the pulse, the event was identified as *harness deployment*. The sequential step-count and harness-deployment events were included in further analyses, if they occurred within the observed segment of the perturbation pulse.

To examine the volunteer’s response time as a dependent variable of the pulse type and direction as factors, two-way repeated measures ANOVA analyses were used. ANOVAs of 3 × 2 design were performed for the acceleration pulses (*Acc1-J1*, *Acc1-J2*, *Acc2-J1*) and directions (forwards, backwards), while a 2 × 2 design was used for the braking pulses (*Br1*, *Br2*) and the two directions. Dependent variables for the ANOVAs were the contact-off time, the swing-time and the muscle onset latency for each of the muscles measured. Prior to the ANOVAs, Grubb’s test and Shapiro-Wilk’s test were used to detect outliers and to test the normality. The sphericity of the datasets was checked with Mauchly’s test and a Greenhouse-Geisser correction was applied in the case of violation. The Bonferroni method for pairwise comparisons was applied. The significance level was set to 0.05. Additionally, the pulse type and gender (male, female) were considered as factors in the two-way repeated measures ANOVAs of 3 × 2 design for the acceleration pulses and 2 × 2 design for the braking pulses, which were used to test for the differences between the male and the female volunteers in the forward and backward directions. Spearman’s correlation coefficient was used to estimate whether the muscle onset latency, contact-off time and swing time were correlated with the volunteer’s body mass and the height of the centre of mass. Statistical analyses were conducted in OriginPRO 2019b (Northampton, MA, United States). The numbers of compensatory steps and harness deployments for each pulse configuration were analysed. In this approach no statistical analysis was conducted, but separate tables were created for the male and female subjects in order to identify the overall response of the volunteers to the pulse type.

## Results

**FIGURE 2:**
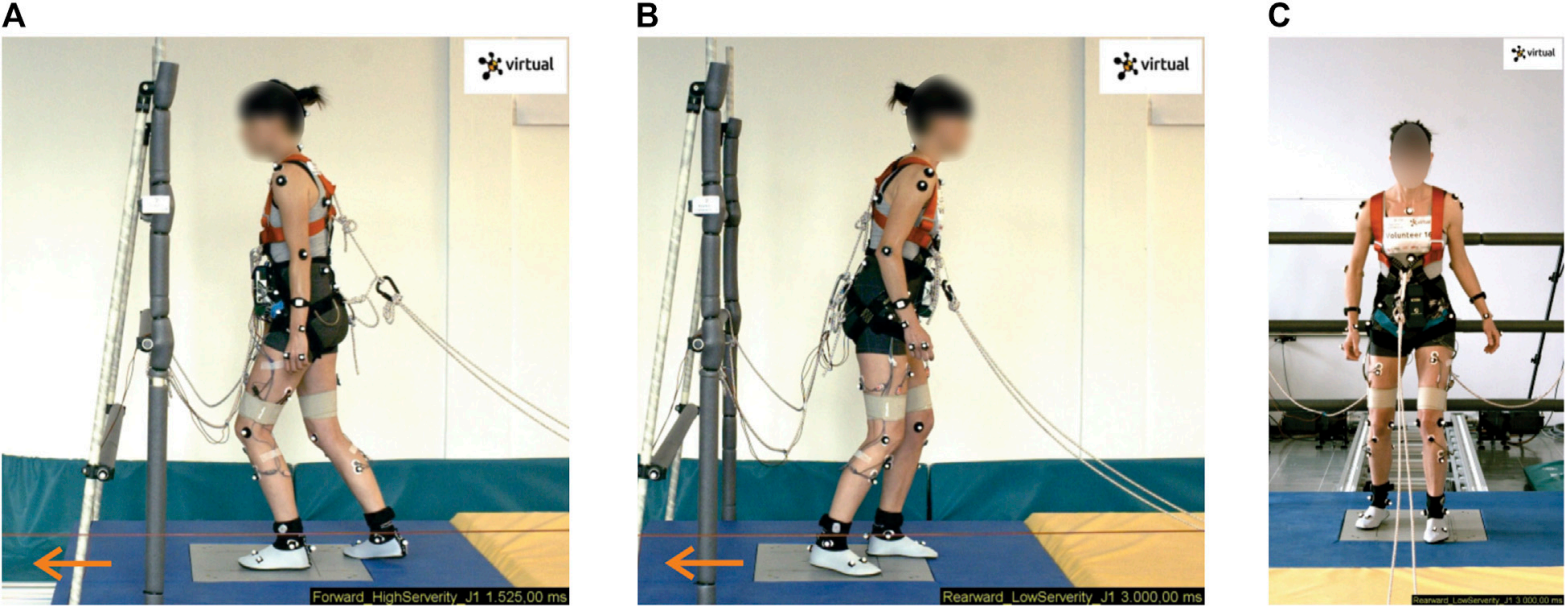
Forward and backward orientation of the volunteers studied in the sled tests. The moving platform induced backward stepping when the volunteers were facing forwards **(A)**, and forward stepping when facing backwards **(B, C)**. The volunteer depicted (Volunteer 16) shows a typical kinematic response during the stepping strategy.

Eleven volunteers finished a complete set of tests with five different pulses in the forward (Figure 2A) and backward (Figures 2B,C) directions, while seven volunteers repeated at least one of the tests. For six volunteers, the higher severity pulses were omitted due to safety considerations. In total, 223 tests were included in the analysis, out of 238 tests conducted with 24 volunteers. More than half (57%) of the volunteers needed at least one compensatory step to maintain their balance for the 1.0 m/s^2^ braking pulse (*Br1*) when facing in the direction of the sled travel and almost all stepped when backward-facing (Table 3). The safety harness was deployed extensively for the *Acc2-J1* profile, again with higher rates in the backward-facing direction. Table 3 shows the general responses to all profiles in both directions.

**TABLE 3:** Percentage of sequential steps (1st–4th) during balance recovery and the percentage of harness deployments for the forward- and backward-facing volunteers (Males/Females).

Profile name	Forwards					Backwards				
	1st	2nd	3rd	4th	Harness	1st	2nd	3rd	4th	Harness
	%	%	%	%	%	%	%	%	%	%
*Br1*	57	52	30	9	4	96	70	57	30	9
M + F	58	58	25	17	8	100	50	58	17	17
M	55	45	36	0	0	91	91	55	45	0
F										
*Br2*	100	82	71	24	0	100	100	83	39	11
M + F	100	73	64	18	0	100	100	91	36	9
M	100	100	83	33	0	100	100	71	43	14
F										
*Acc1-J1*	100	100	67	54	21	100	92	79	33	21
M + F	100	100	69	46	15	100	85	69	23	23
M	100	100	64	64	27	100	100	91	45	18
F										
*Acc1-J2*	100	83	70	48	17	100	78	43	17	22
M + F	100	77	62	23	15	100	75	25	8	25
M	100	90	80	80	20	100	82	64	27	18
F										
*Acc2-J1*	100	100	92	46	75	100	100	88	50	88
M + F	100	100	85	31	69	100	100	92	46	92
M	100	100	100	64	82	100	100	82	55	82
F										

Four outliers were detected with Grubb’s test and removed from the datasets for the contact-off time; Shapiro-Wilk’s test rejected a normal distribution for *Acc1-J1* forwards. Mauchly’s test showed no violations of sphericity for the datasets. For the swing time, two outliers were found and removed; normal distribution was rejected for *Br1* forwards and *Acc1-J2* backwards. The analysis yielded significant main effects of pulse (F (2,38) = 94.3, *p* < 0.001) and direction (F (1,19) = 56.3, *p* < 0.001) on the contact-off time, while the interaction effect of the pulse and direction was not significant (F (2,38) = 0.16, *p* = 0.851). The contact-off time in *Acc1-J1* and *Acc2-J1* was longer than in *Acc1-J2* in both directions (*p* < 0.001). However, no significant difference was found between the contact-off time in *Acc1-J1* and *Acc2-J1*. For the braking pulses, the analysis showed a significant effect of the pulse (F (1,6) = 808.3, *p* < 0.001), with the contact-off time in *Br2* being shorter in both directions. For the acceleration pulses, a significant main effect on swing time was found for the direction (F (1,20) = 10.21, *p* = 0.005), but not for the pulse (F (2,40) = 0.77, *p* = 0.469). The swing time was shorter in the forward direction *Acc1-J1* (*p* = 0.011). For the braking pulses, no significant effects of the pulse or the direction were found. No significant effect of gender on the contact-off and swing time was found.

**FIGURE 3:**
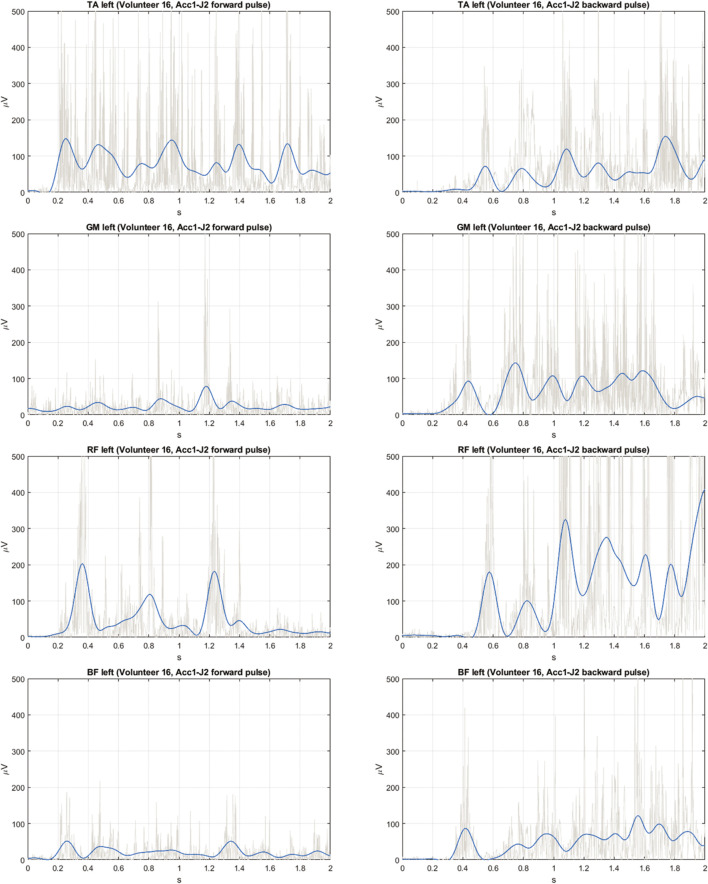
Exemplary EMG signals measured on leg muscles (raw—grey, filtered—blue) for the *Acc1-J2* pulse in forward direction **(left column)** and backward direction **(right column)**; TA, tibialis anterior; GM, gastrocnemius medialis; RF, rectus femoris; BF, biceps femoris.

In 17% of the EMG signals recorded, the onset detected was corrected manually, while in 5% it was not possible to estimate the muscle onset. For each of the eight muscles analysed, 10 datasets on the onset latencies were collected for the five perturbation pulses and two directions. Grubb’s test detected 25 outliers that were removed from the EMG datasets. A Shapiro-Wilk test showed that the assumption of a normal distribution was not met in 29 out of 80 datasets. Based on a further examination of those cases by means of quantile-quantile plots, it was decided to continue with the analysis on the original data-sets without a transformation. An example of the EMG signals recorded on the lower leg muscles is depicted in Figure 3.

**FIGURE 4:**
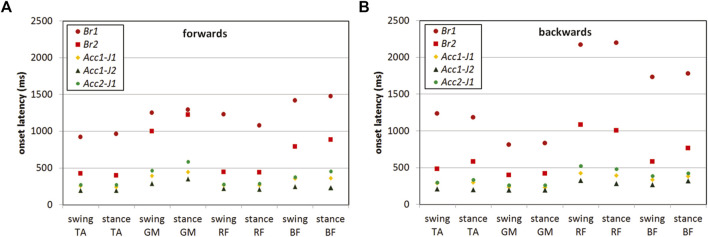
Average EMG onset latencies for the muscles of the swing leg and the stance leg for each pulse type in forward **(A)** and backward **(B)** direction; TA, tibialis anterior; GM, gastrocnemius medialis; RF, rectus femoris; BF, biceps femoris. In both directions, shorter onset latencies were observed in the acceleration pulses, compared to the braking pulses. The shortest active muscle response was evoked by *Acc1-J2* pulse.

Similar to the analysis of the step initiation, the average response times for the volunteers were calculated and are presented in Figure 4 and Tables 5, 6. The responses for all the volunteers as well as for the male and female subgroups are provided.

For the braking pulses *Br1* and *Br2*, a significant main effect of the pulse was observed for all the muscles analysed. As expected, the onset latencies were shorter with a higher acceleration magnitude of *Br2*, compared to *Br1* (Tables 5, 6). In addition, the main effect of direction was observed, except for the swing BF (F (1,8) = 0.15, *p* = 0.710, $\eta_{p}^{2}$ = 0.02) and the stance BF (F (1,10) = 0.69, *p* = 0.424, $\eta_{p}^{2}$ = 0.06). Shorter onset latencies in forward-direction pulses were found for the swing TA (*p* = 0.003) and the stance TA (*p* = 0.003), the swing RF (*p* < 0.001) and the stance RF (*p* < 0.001), while the latencies were shorter in the backward-direction pulses for the swing MG (*p* = 0.011) and the stance GM (*p* = 0.001). An interaction effect (pulse × direction) was found for the swing TA (F (1,15) = 7.59, *p* = 0.014, $\eta_{p}^{2}$ = 0.34) and the swing RF (F (1,15) = 5.38, *p* = 0.035, $\eta_{p}^{2}$ = 0.26). Pairwise comparisons showed that the onset latency of the swing TA was shorter in the forwards *Br1* (*p* = 0.005), but not in *Br2*.

For the acceleration pulses *Acc1-J1*, *Acc1-J2*, and *Acc2-J1*, the main effects of the pulse and direction were found for all the muscles analysed, with the exception of a non-significant direction effect for the swing BF (F (1,16) = 0.20, *p* = 0.661, $\eta_{p}^{2}$ = 0.01) and the stance BF (F (1,13) = 0.47, *p* = 0.506, $\eta_{p}^{2}$ = 0.03). The main effect of direction followed the same pattern as in *Br1* and *Br2*, where the onset latencies were shorter in the forward-direction pulses for TA and RF, and in backward-direction pulses for GM. Pairwise comparisons showed that the onset latencies were significantly shorter in *Acc1-J2* than in *Acc1-J1* and *Acc2-J2* for all the muscles except for the stance GM, which was not significantly different from the latency in *Acc1-J1* (*p* = 0.386).

A significant interaction effect pulse × direction was found for the stance TA (F (1.49,26.82) = 6.89, *p* = 0.007, $\eta_{p}^{2}$ = 0.28), the stance GM (F (2,24) = 3.54, *p* = 0.045, $\eta_{p}^{2}$ = 0.23), the swing RF (F (2,36) = 14.63, *p* < 0.001, $\eta_{p}^{2}$ = 0.45), and the stance RF (F (2,32) = 10.62, *p* < 0.001, $\eta_{p}^{2}$ = 0.40). For the stance TA, post-hoc tests showed no significant effect of direction in *Acc1-J2* and no difference between *Acc1-J1* and *Acc2-J1* in forward perturbations. Direction also had no significant effect in *Acc1-J2* for the stance GM (*p* = 0.072). Furthermore, the onset latencies for the stance GM exhibited no differences between the backward-direction pulses, while the difference in the forward-direction pulses was found only between *Acc1-J2* and *Acc2-J1*, with the latter exhibiting a longer onset latency (*p* = 0.001). The onset latencies for the swing RF were not significantly different among the forward-direction pulses, which was also observed for the stance RF, where no direction effect was found in *Acc1-J2* (*p* = 0.279).

In addition to the main effect of the pulse, a significant main effect of the gender of the volunteers was found, with a tendency for a shorter onset latency with the female volunteers in the acceleration forward-direction trials for the stance TA, (F (1,9) = 9.32, *p* = 0.014, $\eta_{p}^{2}$ = 0.51), stance GM (F (1,4) = 40.53, *p* = 0.003, $\eta_{p}^{2}$ = 0.91), and the stance BF (F (1,5) = 7.51, *p* = 0.041, $\eta_{p}^{2}$ = 0.60). Although no significant differences for the contact-off time between the males and the females were observed, the *p*-value was close to the 0.05 significance level (F (1,8) = 5.27, *p* = 0.051, $\eta_{p}^{2}$ = 0.40). For the acceleration backward-direction trials, no significances for the gender-dependent analyses were found.

Significant positive correlations (Spearman’s coefficient r = 0.42–0.69) were found between the body mass and the onset latencies of the TA, RF and BF muscles (Tables 7, 8) in both the forwards and (even though less prevalent) backward-direction trials. Step timings were not found to have any significant correlation with body mass. The height of the centre of gravity was found to be positively correlated with the muscle onset latencies in both the forward- and backward-direction trials (r = 0.47–0.77) for some muscles, as well as for the contact-off and swing times (Tables 9, 10).

## Discussion

We compared the characteristics of the initial muscle and kinematic responses of healthy volunteers subjected to typical balance perturbations that can be experienced by standing passengers on public transport. Based on a literature review and in-house measured data, a set of perturbation pulses was defined to simulate typical bus accelerations and decelerations in a laboratory environment. The severity of the perturbation pulses was targeted to exceed the comfort zone for standing passengers, yet enable an analysis of the passengers’ initial response in typical accelerations and decelerations of public transport, potentially resulting in non-collision incidents. A strong individual variability was observed during the tests: while some of the participants showed a good ability to counteract the perturbation pulses used, others could not be exposed to the more severe perturbations for safety reasons, which also resulted in missing observations that could not be included in the analysis. No signs of the volunteers’ adaptation to the perturbation pulses were observed.

In both directions of travel, the time until the participants initiated the first recovery step was longer for the braking pulses *Br1* and *Br2* than for the acceleration pulses (Table 4). The participants could maintain their balance without recovery stepping in only about half of the trials with the low-severity braking pulse *Br1*, characterized by a very gradual increase of the acceleration magnitude (Table 3), while at least one recovery step was needed in the acceleration pulses. This is in accordance with observations in another study (Schubert et al., 2017), where volunteers had to make recovery stepping when standing freely in a bus and subjected to accelerating and decelerating manoeuvres comparable to the *Acc1-J1* and *Acc1-J2* pulses, while the magnitude of the deceleration phase was between *Br1* and *Br2* pulses. These authors found characteristic patterns of muscle activity similar to the observations in our study and observed a correlation between the jerk and fast compensatory steps, even though the participating volunteers were elderly (age 68.1 ± 5.2). In addition, the current study presents a more detailed analysis of the timing of the muscle activity and the stepping.

**TABLE 4:** Average times for initiation (contact-off time) and duration (swing time) of the first step, where observed (Males/Females, mean ± SD).

*Profile name*	1st step contact-off time		1st step swing time	
	Forwards ms	Backwards ms	Forwards ms	Backwards ms
*Br1*	3,358 ± 434	3,205 ± 441	153 ± 69	166 ± 67
M + F	3,351 ± 447	3,183 ± 434	177 ± 68	173 ± 68
M	3,366 ± 460	3,236 ± 475	125 ± 65	158 ± 68
F				
*Br2* (M + F)	1,259 ± 228	1,239 ± 234	168 ± 58	177 ± 57
M	1,244 ± 186	1,220 ± 263	177 ± 63	181 ± 62
F	1,288 ± 310	1,269 ± 197	152 ± 46	171 ± 54
*Acc1-J1*	541 ± 96	634 ± 89	136 ± 44	171 ± 55
M + F	556 ± 94	641 ± 79	150 ± 41	181 ± 65
M	523 ± 100	626 ± 104	120 ± 43	160 ± 40
F				
*Acc1-J2*	408 ± 29	505 ± 71	147 ± 57	172 ± 44
M + F	418 ± 30	528 ± 65	150 ± 65	183 ± 49
M	393 ± 21	476 ± 69	143 ± 47	160 ± 36
F				
*Acc2-J1*	577 ± 96	672 ± 92	155 ± 54	165 ± 40
M + F	601 ± 90	682 ± 61	173 ± 44	169 ± 48
M	549 ± 98	660 ± 124	133 ± 60	161 ± 29
F				

The pattern of muscle activation was similar for all the pulses, despite being considerably longer for the braking than for the acceleration pulses (Figure 4). In the forward-direction trials, the anterior leg muscles TA and RF preceded the activation of GM and BF, while in the backward-direction trials the sequence was opposite. In both perturbation directions, the leg muscles tended to activate in distal to proximal sequences, which characterizes the ankle strategy, before making a compensatory step.

For acceleration trials, the tests showed shorter onset latencies in *Acc1-J2* with the highest jerk magnitude (11.3 m/s^3^) compared to *Acc1-J1* and *Acc2-J1* (5.6 m/s^3^), implying that the jerk magnitude is the more important factor in the excitation of the active response of the muscles, rather than the acceleration magnitude. This finding is in agreement with observations that the jerk magnitude and the frequency of occurrence significantly influence the comfort and safety, requiring a corrective response from the passengers (Levis, 1978; Brooks et al., 1980). A further comparison of the pulses *Acc1-J1* and *Acc2-J1* having the same jerk magnitude 5.6 m/s^3^ and different acceleration magnitude yielded significantly shorter onset latencies for the stance TA, swing RF, and stance RF in *Acc1-J1* (1.5 m/s^2^) than in *Acc2-J1* (3.0 m/s^2^). A possible reason is that the jerk magnitude in *Acc1-J1* appeared at 0.1 s, but later in *Acc2-J1*, at 0.2 s (Table 1; Figure 1), evoking a more rapid reflex response in *Acc1-J1*, despite the lower acceleration magnitude. Following the muscle activation, the contact-off time of the first step was significantly shorter with a higher jerk magnitude, but similar with different acceleration magnitudes of the pulses. Hence, a higher acceleration magnitude of a perturbation profile might not necessarily evoke faster recovery stepping within the range of magnitudes tested.

Backward stepping in response to a forward motion of the platform was consistently faster than forward stepping, which can most likely be attributed to the asymmetry of the human body in the sagittal plane, resulting in different motion patterns for forward and backward displacements (Runge et al., 1999). In a study of young adult volunteers (Maki et al., 1996; Maki and McIlroy, 1997), a contact-off time of 409 ± 77 ms after the pulse was initiated and a foot-swing duration of 141 ± 69 ms were reported for backward perturbations, compared to a shorter contact-off time of 368 ± 85 ms and a foot-swing duration of 149 ± 63 ms in forwards perturbations with a 300-ms square acceleration pulse and 0.18-m linear displacements. Although the perturbation profiles used there (Maki et al., 1996; Maki and McIlroy, 1997) differed in duration and displacements from the present study, the contact-off times and swing duration for the first recovery step were comparable for the case of the acceleration pulses applied (Table 4), which could be attributed to the initial jerk of the square pulse, but were longer than the step preparation time of 150–160 ms assumed for the inverted pendulum model (Vallée et al., 2015; Aftab et al., 2016).

For the braking pulses applied, the muscle onset latencies and contact-off time of the first step were longer than for the acceleration pulses, but did not change with the direction of travel. This was present in particular for *Br1*, where the volunteers applied non-stepping as well as stepping strategies to recover their balance, implying larger between-subject variations of the active response, possibly combining reflexive and voluntary reactions. However, the percentage of participants who took at least one recovery step was higher in the backward-direction trials (Table 3), which is in agreement with estimations of the single-step threshold being about 1.0 m/s^2^ for the forward direction and lower for the backward direction, 0.7 m/s^2^ (de Kam et al., 2017).

The rate of harness deployment, indicating excessive whole-body displacement, was greater when travelling backwards than forwards and particularly high in the *Acc2-J1* pulse, 88% (Table 3). An acceleration magnitude of 3.0 m/s^2^ caused almost 90% of the backward-facing participants to fall into the harness, compared to 21% in *Acc1-J1* (which had the same jerk level, but only half the acceleration magnitude). These findings cannot be directly compared to previous studies due to the different setup and design of the safety system, but the sharp rise of the harness deployment between 1.5 and 3.0 m/s^2^ acceleration magnitude confirms the threshold levels of 1.0–1.8 m/s^2^, as recommended for public transport (De Graaf and Van Weperen, 1997; Szturm and Fallang, 1998; Karekla and Tyler, 2018; Karekla and Fang, 2021). The percentage of harness deployment was low in the trials with the *Br2* pulse, even though it was identified as the most challenging to participants during pre-tests and was not used for the volunteers during the tests who raised potential safety concerns. The likely reason was that the sled-stopping segment of *Br2* yielded a magnitude of 3.5 m/s^2^ due to the setup design limitations (Figure 1), which caused the safety concerns, while the volunteers’ response was observed during the rise segment of the pulse only. In 24% of the forward-direction trials and 44% of the backward-direction trials, the harness was deployed after the rise time of *Br2* pulse ended (2.2 s, Table 1), before the stopping of the sled had to be initiated due to the operational limits of the setup. Compared to a free-standing posture, the use of handrails and vertical bars substantially increases the possibility of the standing passengers keeping their balance (Robert et al., 2007a; Sarraf et al., 2014; Schubert et al., 2017). However, if public transport must accommodate free-standing passengers, the vehicle acceleration and braking actions should be such that they minimize the risk of these passengers losing their balance.

The initial response to the perturbations followed the same pattern for the male and female volunteers, although the results collected imply a faster response from the female volunteers (Tables 4–6). Furthermore, the muscle onset latency, contact-off time, and swing time were found to be correlated with the body-mass distribution (Tables 7–10). Hence, the lower body mass and the lower height of the centre of mass of the female volunteers (Table 2) could contribute to their faster response, particularly with the acceleration profiles applied that tend to evoke a mechanical response in the inverted-pendulum manner, possibly triggering sensory feedback and muscle activation faster to recover balance (Winter, 1995; Costello et al., 2012; Aftab et al., 2016; Le Mouel and Brette, 2019). Similar observations were reported in other studies comparing male and female volunteers (Maki et al., 1996; Karekla and Fang, 2021).

**TABLE 5:** Descriptive statistics on EMG onset latencies in forward-direction trials (Males/Females, mean ± SD).

Profile name	Swing leg				Stance leg			
	TA	GM	RF	BF	TA	GM	RF	BF
	ms	ms	ms	ms	ms	ms	ms	ms
*Br1*	919 ± 261	1,251 ± 496	1,228 ± 584	1,419 ± 514	963 ± 256	1,291 ± 457	1,079 ± 502	1,475 ± 648
M + F	991 ± 224	1,274 ± 471	1,254 ± 691	1,546 ± 629	967 ± 245	1,161 ± 561	1,090 ± 624	1,451 ± 807
M	834 ± 287	1,226 ± 550	1,194 ± 442	1,245 ± 229	959 ± 280	1,394 ± 351	1,064 ± 355	1,594 ± 427
F								
*Br2*	421 ± 143	999 ± 320	445 ± 117	790 ± 352	396 ± 97	1,222 ± 326	440 ± 94	886 ± 378
M + F	434 ± 175	1,132 ± 226	444 ± 153	865 ± 383	395 ± 116	1,331 ± 293	436 ± 117	979 ± 365
M	401 ± 75	809 ± 353	446 ± 42	672 ± 283	398 ± 66	1,049 ± 329	447 ± 46	754 ± 383
F								
*Acc1-J1*	254 ± 27	392 ± 153	266 ± 37	357 ± 101	238 ± 42	445 ± 207	267 ± 25	359 ± 122
M + F	265 ± 24	457 ± 171	274 ± 41	392 ± 116	259 ± 25	557 ± 192	269 ± 21	395 ± 127
M	241 ± 25	321 ± 94	257 ± 32	318 ± 68	213 ± 45	323 ± 148	264 ± 29	324 ± 111
F								
*Acc1-J2*	195 ± 24	286 ± 88	221 ± 41	248 ± 78	193 ± 28	349 ± 127	208 ± 15	231 ± 66
M + F	204 ± 29	312 ± 105	238 ± 50	263 ± 104	208 ± 16	386 ± 123	214 ± 12	238 ± 89
M	184 ± 12	257 ± 58	203 ± 16	233 ± 33	176 ± 29	290 ± 118	201 ± 15	222 ± 18
F								
*Acc2-J1*	270 ± 27	465 ± 123	278 ± 37	376 ± 90	271 ± 27	584 ± 227	286 ± 23	455 ± 206
M + F	275 ± 31	493 ± 120	279 ± 47	408 ± 104	280 ± 27	657 ± 187	290 ± 25	519 ± 212
M	265 ± 22	432 ± 124	276 ± 24	341 ± 56	261 ± 25	512 ± 249	282 ± 22	390 ± 186
F								

TA, tibialis anterior; GM, gastrocnemius medialis; RF, rectus femoris; BF, biceps femoris.

**TABLE 6:** Descriptive statistics on EMG onset latencies in backward-direction trials (Males/Females, mean ± SD).

Profile name	Swing leg				Stance leg			
	TA	GM	RF	BF	TA	GM	RF	BF
	ms	ms	ms	ms	ms	ms	ms	ms
*Br1*	1,236 ± 346	809 ± 315	2,172 ± 943	1733 ± 785	1,184 ± 273	829 ± 228	2,195 ± 846	1777 ± 988
M + F	1,214 ± 313	728 ± 296	2,334 ± 920	1809 ± 814	1,221 ± 298	787 ± 273	2,275 ± 797	1905 ± 999
M	1,265 ± 401	889 ± 329	1937 ± 980	1,675 ± 787	1,132 ± 239	860 ± 193	2091 ± 939	1,649 ± 1,008
F								
*Br2*	480 ± 133	395 ± 83	1,081 ± 264	578 ± 132	581 ± 127	416 ± 104	1,006 ± 325	765 ± 331
M + F	488 ± 124	424 ± 80	1,071 ± 283	630 ± 148	624 ± 125	402 ± 109	949 ± 320	773 ± 319
M	466 ± 161	349 ± 70	1,098 ± 252	502 ± 41	509 ± 101	435 ± 101	1,095 ± 336	755 ± 373
F								
*Acc1-J1*	288 ± 64	236 ± 49	425 ± 135	333 ± 58	297 ± 45	232 ± 45	393 ± 109	383 ± 127
M + F	296 ± 47	235 ± 65	437 ± 96	327 ± 60	310 ± 54	229 ± 50	374 ± 95	395 ± 121
M	279 ± 82	237 ± 22	410 ± 175	340 ± 59	284 ± 27	235 ± 41	418 ± 125	370 ± 137
F								
*Acc1-J2*	211 ± 34	195 ± 14	326 ± 109	267 ± 49	201 ± 43	194 ± 20	280 ± 71	321 ± 159
M + F	219 ± 17	202 ± 9	359 ± 110	295 ± 42	191 ± 52	195 ± 27	296 ± 71	336 ± 179
M	201 ± 48	187 ± 14	278 ± 93	237 ± 37	216 ± 24	193 ± 10	257 ± 67	303 ± 135
F								
*Acc2-J1*	296 ± 58	260 ± 25	521 ± 136	386 ± 97	336 ± 72	259 ± 36	481 ± 137	422 ± 127
M + F	297 ± 67	269 ± 26	521 ± 124	408 ± 95	365 ± 81	273 ± 40	512 ± 139	446 ± 94
M	295 ± 49	249 ± 20	520 ± 157	359 ± 98	302 ± 44	242 ± 23	436 ± 130	397 ± 154
F								

TA, tibialis anterior; GM, gastrocnemius medialis; RF, rectus femoris; BF, biceps femoris.

**TABLE 7:** Correlations between body mass and EMG onset latencies, contact-off time and swing time in forward-direction trials.

Profile name	Swing leg				Stance leg			
	TA	GM	RF	BF	TA	GM	RF	BF	Contact-off time	Swing time
*Br1*	0.10	−0.23	−0.16	0.19	−0.04	−0.22	−0.16	−0.16	−0.15	0.51
*Br2*	**0.61**	0.35	**0.43**	**0.42**	**0.64**	0.38	0.29	0.40	−0.10	−0.03
*Acc1-J1*	**0.57**	0.14	**0.48**	0.21	**0.69**	0.29	**0.63**	**0.53**	0.08	−0.37
*Acc1-J2*	0.09	0.04	0.09	0.43	0.28	0.28	−0.08	0.41	0.15	0.18
*Acc2-J1*	−0.11	0.35	−0.22	**0.47**	−0.18	0.50	−0.10	0.40	−0.01	0.15

TA, tibialis anterior; GM, gastrocnemius medialis; RF, rectus femoris; BF, biceps femoris.

Values in bold indicate statistical significance.

**TABLE 8:** Correlations between body mass and EMG onset latencies, contact-off time and swing time in backward-direction trials.

Profile name	Swing leg				Stance leg			
	TA	GM	RF	BF	TA	GM	RF	BF	Contact-off time	Swing time
*Br1*	−0.40	−0.23	0.08	0.07	0.03	0.08	0.04	−0.03	−0.15	−0.13
*Br2*	0.13	0.35	0.16	0.19	0.16	−0.06	−0.12	0.19	0.10	0.17
*Acc1-J1*	0.29	0.14	0.39	**0.66**	0.00	0.35	0.10	−0.07	0.11	0.12
*Acc1-J2*	−0.08	0.04	0.19	0.16	0.12	0.38	0.37	−0.03	0.16	0.03
*Acc2-J1*	−0.10	0.35	−0.17	0.23	0.21	−0.31	−0.21	−0.09	−0.17	0.34

TA, tibialis anterior; GM, gastrocnemius medialis; RF, rectus femoris; BF, biceps femoris.

Values in bold indicate statistical significance.

**TABLE 9:** Correlations between centre of gravity height and EMG onset latencies, contact-off time and swing time in forward-direction trials.

Profile name	Swing leg				Stance leg			
	TA	GM	RF	BF	TA	GM	RF	BF	Contact-off time	Swing time
*Br1*	0.29	0.40	0.23	0.18	−0.17	0.26	0.30	−0.05	0.06	0.09
*Br2*	**0.51**	**0.52**	0.20	**0.47**	**0.67**	**0.49**	0.20	0.41	0.19	0.39
*Acc1-J1*	**0.57**	0.15	**0.54**	0.28	**0.77**	0.19	**0.60**	0.36	**0.44**	−0.01
*Acc1-J2*	0.22	0.08	0.05	0.39	0.35	0.12	0.33	**0.66**	**0.51**	0.29
*Acc2-J1*	0.34	0.23	0.18	0.46	0.12	0.34	0.15	0.31	0.13	0.30

TA, tibialis anterior; GM, gastrocnemius medialis; RF, rectus femoris; BF, biceps femoris.

Values in bold indicate statistical significance.

**TABLE 10:** Correlations between centre of gravity height and EMG onset latencies, contact-off time and swing time in backward-direction trials.

Profile name	Swing leg				Stance leg			
	TA	GM	RF	BF	TA	GM	RF	BF	Contact-off time	Swing time
*Br1*	−0.12	−0.39	0.44	0.06	0.19	−0.21	**0.45**	−0.12	0.06	0.19
*Br2*	0.09	0.08	0.41	0.01	0.41	−0.02	0.07	**0.57**	**0.61**	**0.59**
*Acc1-J1*	0.32	0.43	**0.66**	**0.51**	−0.21	0.22	0.32	0.14	**0.62**	0.31
*Acc1-J2*	0.05	0.19	**0.65**	0.28	0.32	0.16	**0.64**	0.40	0.36	**0.51**
*Acc2-J1*	0.05	0.20	−0.08	0.03	0.29	−0.51	0.19	−0.31	0.02	0.27

TA, tibialis anterior; GM, gastrocnemius medialis; RF, rectus femoris; BF, biceps femoris.

Values in bold indicate statistical significance.

However, in order to provide more definite conclusions that could be applied to gender-specific HBM modelling, increasing the number of test subjects would provide more input data for the analysis methods used. The influence of age on balance recovery has not been examined in this study. It is reported in the literature that younger adults are capable of shorter step-initiation and completion times, while the elderly can respond as fast as younger populations in reflexive stepping (which is generally faster than voluntary stepping) (Rogers et al., 2003; Tokuno et al., 2010). Therefore, the outcomes of the present study with volunteers aged 33.8 ± 9.2 might also be representative for elderly passengers exposed to forward and backward perturbations.

Only the initial response of free-standing occupants to balance perturbations in the anterior-posterior direction was considered in the present study, which offers the smallest base of support to react against the applied loads and might lead to large body displacements, increasing the risk of impacts. Moreover, it is a suitable choice for the initial HBM standing position, which can be modified to other postures that might be used by the occupants of a bus. Passengers oriented laterally with respect to the perturbation might exhibit better resistance to perturbations due to a larger base of support. However, elderly people (above the age of 65–70) have been found to have a higher risk of falling and injury, tending to perform cross-over steps more often compared to lateral sidesteps that are used by younger adults (Maki et al., 2000; Mille et al., 2013; Borelli et al., 2019). Such complex balance strategies were out of the scope of this study, but future research should investigate occupant postures with different foot positions and study the use of handrails and vertical bars, as well as measuring other muscles that might contribute to balance recovery (Oude Nijhuis et al., 2010). The study was conducted in a laboratory setting, offering a high level of control over the test parameters. Yet, despite the preventive measures taken, the possibility of the volunteers getting habituated to the perturbation pulses cannot be entirely excluded. Furthermore, due to the safety aspect and technical limitations it was not possible to precisely replicate the environment of the bus and the perturbations that could be experienced by the bus passengers.

The results of this study suggest, as a starting point, that the peak accelerations of a bus should be below 1.5 m/s^2^ during the journey, while the jerk magnitudes used in the study were higher (over 5 m/s^3^) than recommended for comfortable travel, but still allowed the volunteers to recover their balance effectively with the room for compensatory stepping provided. For the braking event, the deceleration should be below 1.0 m/s^2^. These values are based on volunteer tests with young volunteers. It is assumed that these values would need to be adjusted downwards when established for the range of the population using public transport. This work is still to be done. Once established, it would serve to define virtual testing procedures for public transport vehicles, providing an efficient approach to assessing the design and operational characteristics of the vehicles, as well as guidance both for bus drivers and for prescribing the take-off and braking of autonomous vehicles.

## Conclusion

This study investigated the response of standing passengers on public transport to balance perturbations, establishing a reference set of experimental data for estimating safe operating envelopes. The focus was on muscle-activation patterns and the kinematic response to forwards and backwards platform translations. By testing several perturbation profiles based on real-world recorded data in a controlled laboratory setup, it was possible to estimate the neuromuscular response in transition from fixed-support strategies to single or multiple stepping strategies for balance recovery. The data collected provides a basis for further developing tools to improve passenger safety and public transit functions, including bus manoeuvring.

It was shown that the shape, magnitude and duration of the perturbation profile significantly affect the initial response of erect passengers. A higher jerk evoked faster muscle activity and recovery steps, which could be expected in both younger and older healthy adults. Bus acceleration can induce a higher risk of the passenger falling than braking due to the higher jerk content, as observed in the pulses used in this study. Greater passenger motion can also arise from longer perturbation durations as experienced in moderate accelerating and braking events. Different combinations of perturbation characteristics elicit a variety of balance-recovery responses. A combination of jerk and acceleration magnitude should be considered when analysing the balance response in virtual testing with generic perturbations. In addition, the study results imply that gender-specific modelling might improve the biofidelity of human body models for simulating the balance recovery of standing passengers in non-collision incidents of public transport vehicles, as gender-specific differences for the muscle onset times were observed. Future research should provide a larger sample of the volunteers subjected to a greater variety of load cases.

## Data Availability

The raw data supporting the conclusions of this article will be made available by the authors, without undue reservation.

## References

[B1] Aftab Z. Robert T. Wieber P.-B. (2016). Balance Recovery Prediction with Multiple Strategies for Standing Humans. PLoS ONE. 11 (3), e0151166. 10.1371/journal.pone.0151166 26974820 PMC4790971

[B2] Albertsson P. Falkmer T. (2005). Is There a Pattern in European Bus and Coach Incidents? A Literature Analysis with Special Focus on Injury Causation and Injury Mechanisms. Accid. Anal. Prev. 37 (2), 225–233. 10.1016/j.aap.2004.03.006 15667808

[B3] Bair W.-N. Prettyman M. G. Beamer B. A. Rogers M. W. (2016). Kinematic and Behavioral Analyses of Protective Stepping Strategies and Risk for Falls Among Community Living Older Adults. Clin. Biomech. 36, 74–82. 10.1016/j.clinbiomech.2016.04.015 PMC5568553 27228075

[B4] Barnes J. Morris A. Welsh R. Summerskill S. Marshall R. Kendrick D. (2016). Injuries to Older Users of Buses in the UK. Public Transp. 8 (1), 25–38. 10.1007/s12469-015-0113-8

[B5] Björnstig U. Bylund P.-O. Albertsson P. Falkmer T. Björnstig J. Petzäll J. (2005). Injury Events Among Bus and Coach Occupants. IATSS Res. 29 (1), 79–87. 10.1016/S0386-1112(14)60121-7

[B6] Blenkinsop G. M. Pain M. T. G. Hiley M. J. (2017). Balance Control Strategies during Perturbed and Unperturbed Balance in Standing and Handstand. R. Soc. Open Sci. 4 (7), 161018. 10.1098/rsos.161018 28791131 PMC5541526

[B7] Borrelli J. Creath R. A. Pizac D. Hsiao H. Sanders O. P. Rogers M. W. (2019). Perturbation-evoked Lateral Steps in Older Adults: Why Take Two Steps when One Will Do? Clin. Biomech. (Bristol, Avon). 63, 41–47. 10.1016/j.clinbiomech.2019.02.014 PMC6501204 30825811

[B8] Brooks B. M. Edwards H. M. Fraser C. R. Levis J. A. Johnson M. A. (1980). Passenger Problems on Moving Buses. Supplementary Report 520. Crowthorn, Berkshire: Crowthorn: Transport and road research laboratory.

[B9] Čamernik J. Potocanac Z. Peternel L. Babič J. (2016). Holding a Handle for Balance during Continuous Postural Perturbations-Immediate and Transitionary Effects on Whole Body Posture. Front. Hum. Neurosci. 10, 486. 10.3389/fnhum.2016.00486 27725798 PMC5035747

[B10] Carlsson A. Linder A. Davidsson J. Hell W. Schick S. Svensson M. (2011). Dynamic Kinematic Responses of Female Volunteers in Rear Impacts and Comparison to Previous Male Volunteer Tests. Traffic Inj. Prev. 12 (4), 347–357. 10.1080/15389588.2011.585408 21823943

[B11] Carpenter M. G. Thorstensson A. Cresswell A. G. (2005). Deceleration Affects Anticipatory and Reactive Components of Triggered Postural Responses. Exp. Brain Res. 167, 433–445. 10.1007/s00221-005-0049-3Costello 16041500

[B12] Costello K. E. Matrangola S. L. Madigan M. L. (2012). Independent Effects of Adding Weight and Inertia on Balance during Quiet Standing. Biomed. Eng. Online. 11, 20. 10.1186/1475-925X-11-20 22507125 PMC3416723

[B13] Cyr M.-A. Smeesters C. (2009). Kinematics of the Threshold of Balance Recovery Are Not Affected by Instructions Limiting the Number of Steps in Younger Adults. Gait & Posture. 29 (4), 628–633. 10.1016/j.gaitpost.2009.01.011 19243948

[B14] de Kam D. Roelofs J. M. B. Bruijnes A. K. B. D. Geurts A. C. H. Weerdesteyn V. (2017). The Next Step in Understanding Impaired Reactive Balance Control in People with Stroke: the Role of Defective Early Automatic Postural Responses. Neurorehabil. Neural Repair. 31 (8), 708–716. 10.1177/1545968317718267 28691582 PMC5714159

[B15] Elvik R. (2019). Risk of Non-collision Injuries to Public Transport Passengers: Synthesis of Evidence from Eleven Studies. J. Transport Health. 13, 128–136. 10.1016/j.jth.2019.03.017

[B16] Graaf B. D. Van Weperen W. (1997). The Retention of Blance: An Exploratory Study into the Limits of Acceleration the Human Body Can Withstand without Losing Equilibrium. Hum. Factors. 39 (1), 111–118. 10.1518/001872097778940614 9302883

[B17] Halpern P. Siebzehner M. I. Aladgem D. Sorkine P. Bechar R. (2005). Non-collision Injuries in Public Buses: a National Survey of a Neglected Problem. Emerg. Med. J. 22 (2), 108–110. 10.1136/emj.2003.013128 15662059 PMC1726661

[B18] Hoberock L. L. (1976). A Survey of Longitudinal Acceleration comfort Studies in Ground Transportation Vehicles. Austin TX: The University of Texas. Research Report 40.

[B19] Hodges P. Bui B. H. (1996). A Comparison of Computer-Based Methods for the Determination of Onset of Muscle Contraction Using Electromyography. Electroencephalogr. Clin. Neurophysiol. 101, 511–519. 10.1016/s0013-4694(96)95190-5 9020824

[B20] Horak F. B. Nashner L. M. (1986). Central Programming of Postural Movements: Adaptation to Altered Support-Surface Configurations. J. Neurophysiol. 55 (6), 1369–1381. 10.1152/jn.1986.55.6.1369 3734861

[B21] Hsiao-Wecksler E. T. Robinovitch S. N. (2007). The Effect of Step Length on Young and Elderly Women's Ability to Recover Balance. Clin. Biomech. 22, 574–580. 10.1016/j.clinbiomech.2007.01.013 17391819

[B22] Hwang S. Tae K. Sohn R. Kim J. Son J. Kim Y. (2009). The Balance Recovery Mechanisms against Unexpected Forward Perturbation. Ann. Biomed. Eng. 37 (8), 1629–1637. 10.1007/s10439-009-9717-y 19472056

[B23] Jin X. Begeman P. Board D. Pline K. Shen M. Sundararajan S. Yang K. H. (2019). “Comparison of Small Female and Mid-sized Male PMHS Response with an Inflatable Seatbelt System during Drontal Impacts,” in 2019 International Research Council on Biomechanics of Injury (IRCOBI) Conference, Florence (Italy), 11–13 September 2019. IRC-19-21. 10.2118/194709-ms

[B24] Karekla X. Fang C. (2021). Upper Body Balancing Mechanisms and Their Contribution to Increasing Bus Passenger Safety. Saf. Sci. 133, 105014. 10.1016/j.ssci.2020.105014

[B25] Karekla X. Tyler N. (2018). Reducing Non-collision Injuries Aboard Buses: Passenger Balance whilst Walking on the Lower Deck. Saf. Sci. 105, 128–133. 10.1016/j.ssci.2018.01.021

[B26] Kendrick D. Drummond A. Logan P. Barnes J. Worthington E. (2015). Systematic Review of the Epidemiology of Non-collision Injuries Occurring to Older People during Use of Public Buses in High Income Countries. J. Transport Health. 2 (3), 394–405. 10.1016/j.jth.2015.06.002

[B27] Kirchner M. Schubert P. Haas C. T. (2014). Characterisation of Real-World Bus Acceleration and Deceleration Signals. Jsip 05, 8–13. 10.4236/jsip.2014.51002

[B28] Kirk A. Grant R. Bird R. (2003). “Passenger Casualties in Non-collision Incidents on Buses and Coaches in Great Britain,” in 18th International Technical Conference on the Enhanced Safety of Vehicles (ESV), Nagoya, Japan, 19–22 May 2003. Paper No. 296.

[B29] Koushyar H. Bieryla K. A. Nussbaum M. A. Madigan M. L. (2019). Age-related Strength Loss Affects Non-stepping Balance Recovery. PLoS ONE. 14 (1), e0210049. 10.1371/journal.pone.0210049 30657760 PMC6338353

[B30] Kühn W. (2013). Fundamentals of Road Design. Southampton: WIT Press. 10.5772/55837

[B31] Le Mouel C. Brette R. (2019). Anticipatory Coadaptation of Ankle Stiffness and Sensorimotor Gain for Standing Balance. Plos Comput. Biol. 15 (11), e1007463. 10.1371/journal.pcbi.1007463 31756199 PMC6897426

[B32] Levis J. A. (1978). The Seated Bus Passenger - a Review. Appl. Ergon. 9 (3), 143–150. 10.1016/0003-6870(78)90004-2 15677264

[B33] Maki B. E. Edmondstone M. A. McIlroy W. E. (2000). Age-related Differences in Laterally Directed Compensatory Stepping Behavior. J. Gerontol. Ser. A: Biol. Sci. Med. Sci. 55 (5), M270–M277. 10.1093/gerona/55.5.M270 10819317

[B34] Maki B. E. McIlroy W. E. Perry S. D. (1996). Influence of Lateral Destabilization on Compensatory Stepping Responses. J. Biomech. 29 (3), 343–353. 10.1016/0021-9290(95)00053-4 8850640

[B35] Maki B. E. McIlroy W. E. (1997). The Role of Limb Movements in Maintaining Upright Stance: the "Change-In-Support" Strategy. Phys. Ther. 77 (5), 488–507. 10.1093/ptj/77.5.488 9149760

[B36] Mille M.-L. Johnson-Hilliard M. Martinez K. M. Zhang Y. Edwards B. J. Rogers M. W. (2013). One Step, Two Steps, Three Steps More … Directional Vulnerability to Falls in Community-Dwelling Older People Directional Vulnerability to Falls in Community-Dwelling Older People. J. Gerontol. A. Biol. Sci. Med. Sci. 68 (12), 1540–1548. 10.1093/gerona/glt062 23685768 PMC3814241

[B37] Oude Nijhuis L. B. Allum J. H. J. Valls-Solé J. Overeem S. Bloem B. R. (2010). First Trial Postural Reactions to Unexpected Balance Disturbances: A Comparison with the Acoustic Startle Reaction. J. Neurophysiol. 104 (5), 2704–2712. 10.1152/jn.01080.2009 20810688

[B38] Owings T. M. Pavol M. J. Grabiner M. D. (2001). Mechanisms of Failed Recovery Following Postural Perturbations on a Motorized Treadmill Mimic Those Associated with an Actual Forward Trip. Clin. Biomech. 16 (9), 813–819. 10.1016/S0268-0033(01)00077-8 11714559

[B39] Palacio A. Tamburro G. O’Neill D. Simms C. K. (2009). Non-collision Injuries in Urban Buses-Strategies for Prevention. Accid. Anal. Prev. 41, 1–9. 10.1016/j.aap.2008.08.016 19114131

[B40] Powell J. P. Palacín R. (2015). Passenger Stability within Moving Railway Vehicles: Limits on Maximum Longitudinal Acceleration. Urban Rail Transit. 1 (2), 95–103. 10.1007/s40864-015-0012-y

[B41] Robert T. Beillas P. Maupas A. Verriest J.-P. (2007a). Conditions of Possible Head Impacts for Standing Passengers in Public Transportation: an Experimental Study. Int. J. Crashworthiness. 12 (3), 319–327. 10.1080/13588260701433552

[B42] Robert T. Beillas P. Maupas A. Verriest J.-P. (2007b). “Possible Head Impacts for Standing Passengers in Public Transportation – Influence of an Obstacle on the Passenger Kinematics,” in 2007 International Research Council on Biomechanics of Injury (IRCOBI) Conference, Maastricht, Netherlands, 19-21 September 2007, 393–396.

[B43] Rogers M. W. Johnson M. E. Martinez K. M. Mille M.-L. Hedman L. D. (2003). Step Training Improves the Speed of Voluntary Step Initiation in Aging. Journals Gerontol. Ser. A: Biol. Sci. Med. Sci. 58 (1), M46–M51. 10.1093/gerona/58.1.M46 12560410

[B44] Runge C. F. Shupert C. L. Horak F. B. Zajac F. E. (1999). Ankle and Hip Postural Strategies Defined by Joint Torques. Gait & Posture. 10 (2), 161–170. 10.1016/S0966-6362(99)00032-6 10502650

[B45] Sarraf T. A. Marigold D. S. Robinovitch S. N. (2014). Maintaining Standing Balance by Handrail Grasping. Gait & Posture. 39 (1), 258–264. 10.1016/j.gaitpost.2013.07.117 23948334

[B46] Schubert P. Liebherr M. Kersten S. Haas C. T. (2017). Biomechanical Demand Analysis of Older Passengers in a Standing Position during Bus Transport. J. Transport Health. 4, 226–236. 10.1016/j.jth.2016.12.002

[B47] Silvano A. P. Ohlin M. (2019). Non-collision Incidents on Buses Due to Acceleration and Braking Manoeuvres Leading to Falling Events Among Standing Passengers. J. Transport Health. 14, 100560. 10.1016/j.jth.2019.04.006

[B48] Siman-Tov M. Radomislensky I. Marom I. Kapra O. Peleg K. Bahouth H. (2019). A Nation-wide Study on the Prevalence of Non-collision Injuries Occurring during Use of Public Buses. J. Transport Health. 13, 164–169. 10.1016/j.jth.2019.03.019

[B49] Simoneau M. Corbeil P. (2005). The Effect of Time to Peak Ankle Torque on Balance Stability Boundary: Experimental Validation of a Biomechanical Model. Exp. Brain Res. 165 (2), 217–228. 10.1007/s00221-005-2290-1 15940496

[B50] Szturm T. Fallang B. (1998). Effects of Varying Acceleration of Platform Translation and Toes-Up Rotations on the Pattern and Magnitude of Balance Reactions in Humans. J. Vestib. Res. 8 (5), 381–397. 10.3233/VES-1998-8504 9770656

[B51] Tokuno C. D. Cresswell A. G. Thorstensson A. Carpenter M. G. (2010). Age-related Changes in Postural Responses Revealed by Support-Surface Translations with a Long Acceleration-Deceleration Interval. Clin. Neurophysiol. 121 (1), 109–117. 10.1016/j.clinph.2009.09.025 19903591

[B52] Torres-Oviedo G. Ting L. H. (2007). Muscle Synergies Characterizing Human Postural Responses. J. Neurophysiol. 98 (4), 2144–2156. 10.1152/jn.01360.2006 17652413

[B53] Vallée P. Tisserand R. Robert T. (2015). Possible Recovery or Unavoidable Fall? A Model to Predict the One Step Balance Recovery Threshold and its Stepping Characteristics. J. Biomech. 48 (14), 3905–3911. 10.1016/j.jbiomech.2015.09.024 26602371

[B54] Vasavada A. N. Li S. Delp S. L. (2001). Three-dimensional Isometric Strength of Neck Muscles in Humans. Spine 26 (17), 1904–1909. 10.1097/00007632-200109010-00018 11568704

[B55] Winter D. (1995). Human Balance and Posture Control during Standing and Walking. Gait & Posture. 3 (4), 193–214. 10.1016/0966-6362(96)82849-9

[B56] Zemková E. Kováčiková Z. Jeleň M. Neumannová K. Janura M. (2016). Postural and Trunk Responses to Unexpected Perturbations Depend on the Velocity and Direction of Platform Motion. Physiol. Res. 65, 769–776. 10.33549/physiolres.933177 27429117

[B57] Zhou H. Yuan C. Dong N. Wong S. C. Xu P. (2020). Severity of Passenger Injuries on Public Buses: A Comparative Analysis of Collision Injuries and Non-collision Injuries. J. Saf. Res. 74, 55–69. 10.1016/j.jsr.2020.04.003 32951796

